# Radiomics analysis based on CT for predicting lymph node metastasis and prognosis in duodenal papillary carcinoma

**DOI:** 10.1186/s13244-024-01732-6

**Published:** 2024-06-20

**Authors:** Chao-Tao Tang, Yonghui Wu, Longzhou Jiang, Chun-Yan Zeng, You-Xiang Chen

**Affiliations:** 1 Department of Gastroenterology, Digestive Disease Hospital, The First Affiliated Hospital, Jiangxi Medical College, Nanchang University, Nanchang, China; 2 Postdoctoral Innovation Practice Base, The First Affiliated Hospital of Nanchang University, Nanchang, People’s Republic of China; 3 Jiangxi Clinical Research Center for Gastroenterology, Nanchang, Jiangxi China

**Keywords:** Duodenal papillary carcinoma, Radiomics, Survival, Lymph node metastasis, CT imaging

## Abstract

**Objectives:**

Radiomics has been demonstrated to be strongly associated with TNM stage and patient prognosis. We aimed to develop a model for predicting lymph node metastasis (LNM) and survival.

**Methods:**

For radiomics texture selection, 3D Slicer 5.0.3 software and the least absolute shrinkage and selection operator (LASSO) algorithm were used. Subsequently, the radiomics model, computed tomography (CT) image, and clinical risk model were compared. The performance of the three models was evaluated using receiver operating characteristic (ROC) curves, decision curve analysis (DCA), calibration plots, and clinical impact curves (CICs).

**Results:**

For the LNM prediction model, 224 patients with LNM information were used to construct a model that was applied to predict LNM. According to the CT data and clinical characteristics, we constructed a radiomics model, CT imaging model and clinical model. The radiomics model for evaluating LNM status showed excellent calibration and discrimination in the training cohort (AUC = 0.926, 95% CI = 0.869–0.982) and the validation cohort (AUC = 0.872, 95% CI = 0.802–0.941). DeLong’s test demonstrated that the difference among the three models was significant. Similarly, DCA and CIC showed that the radiomics model has better clinical utility than the CT imaging model and clinical model. Our model also exhibited good performance in predicting survival—in line with the findings of the model built with clinical risk factors.

**Conclusions:**

CT radiomics models exhibited better predictive performance for LNM than models built based on clinical risk characteristics and CT imaging and had comparative clinical utility for predicting patient prognosis.

**Critical relevance statement:**

The radiomics model showed excellent performance and discrimination for predicting LNM and survival of duodenal papillary carcinoma (DPC).

**Key Points:**

- LNM status determines the most appropriate treatment for DPC.
- Our radiomics model for evaluating the LNM status of DPC performed excellently.
- The radiomics model had high sensitivity and specificity for predicting survival, exhibiting great clinical value.

**Graphical Abstract:**

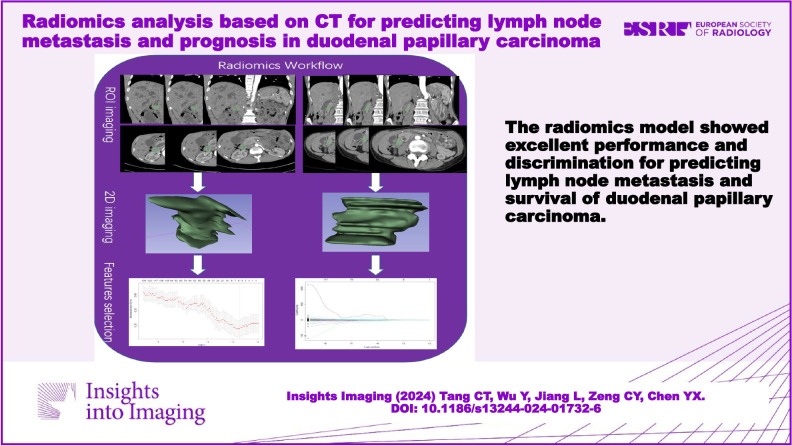

## Introduction

The duodenum is a component of the small intestine that is 5–6 m long, and tumours rarely occur there. Duodenal carcinoma accounts for approximately 50% of small intestinal cancers and approximately 0.3% of gastroenterological cancers [1]. The duodenal papilla is an important site of carcinogenesis because it is formed by a common duct linking the bile duct with the pancreatic duct, the surrounding sphincter muscle, and the papillary bulge of the duodenal mucosa. The greatest function of the papilla is to participate in regulating the secretion of bile and pancreatic fluid; therefore, once tumours occur, patients will experience jaundice and indigestion [2].

Duodenal papillary carcinoma (DPC) usually involves the duodenum, bile ducts, and pancreatic ducts. Previous studies have shown that lesions are mostly located at the common opening of the duodenal papilla [2]. DPCs in the early stage are mistaken for chronic inflammation or benign protuberant lesions due to their small size, as determined by CT, endoscopic examination and other methods. Furthermore, the incidence of small intestinal carcinoma and DPC has increased annually, and the mortality rate has increased by 26% [3]. As many patients are asymptomatic until advanced disease detection through imaging examination, diagnostic delays often occur, leading to a poor prognosis [4]. Previous studies have shown that more than 56% of patients exhibit locally advanced tumours or distant metastases at initial diagnosis, and those with advanced tumours only have a 5-year survival rate of 42.6% [3]. For papilla tumours in situ without lymphovascular invasion (LVI) or lymph node metastasis (LNM), endoscopic papillectomy is safe and curative; however, when tumours exhibit LNM or LVI, surgery is considered the first modality without considering endoscopy [5, 6]. Most studies have suggested that LNM is associated with prognosis, and the incidence of LNM ranges from 22% to 76% [7, 8]. Undoubtedly, the LNM status can determine the method of treatment and affect whether patients can undergo curative resection. Clinically, if patients are identified as having positive LNM, chemotherapy should be administered after surgery or before surgery. Hence, identifying the status of LNMs is crucial for managing patients and predicting the prognosis of DPC patients.

There are few previous studies on the ability of the model to predict LNM and patient prognosis in DPC, and the performance of the model is not desirable [9, 10]. Endoscopic ultrasound and intraductal ultrasonography are also unsatisfactory for detecting LNM, with a sensitivity and specificity of 0.61 and 0.77, respectively [11]. Radiomics is an emerging diagnostic and predictive method that facilitates accurate diagnosis by extensively exploring, predicting, and analysing a vast amount of medical imaging data [12]. Radiomics can identify heterogeneity within tissues and employ automated high-throughput feature extraction algorithms to convert image data into readable quantitative data [13]; this transformation enables healthcare professionals to better comprehend the information embedded in images and apply it in clinical practice. To date, many studies have focused on radiomics for predicting LNM, as well as LVI, and these studies have demonstrated that radiomics has a promising future in clinical practice [14, 15]. However, whether radiomics is an effective tool for individualised prediction of LNM in DPC patients is unknown.

In our study, to establish a new radiomics model based on CT imaging for LNM in the DPC, 224 patients with DPC were enroled between January 2018 and September 2022 for statistical analysis, after which the performance of the model was evaluated internally and externally.

## Methods

### Patient extraction

All patients diagnosed with DPC between January 2018 and September 2022 were obtained from the First Affiliated Hospital of Nanchang University. The inclusion criteria were as follows: (1) diagnosed with DPC based on histological examination, (2) had detailed CT data recorded, and (3) had complete LNM data. We excluded the following patients: (1) patients who did not undergo surgery; (2) patients with incomplete or unavailable imaging data; (3) patients with other severe diseases, such as renal failure and heart failure; and (4) patients receiving neoadjuvant chemotherapy. For patients with missing survival information, we recorded the information via telephone follow-up. All patients were followed up until October 2022. Finally, 224 patients from our centre were randomly assigned to two groups at a 1:1 ratio: the training group (112 patients) and the testing group (112 patients). The study flowchart is shown in Supplementary Fig. 1.

### Definitions of variables

In our study, the following data were collected: (1) clinical characteristics, including age, sex, drinking status, smoking status, survival time, and CT imaging; (2) pathological-related features, including TNM stage, tumour size, and cell differentiation; and (3) serum markers, including CEA and CA199. Patient characteristics are summarised in Table 1. Sex was recorded as male or female, and age was categorised into < 50 and ≥ 50-years-old. Clinical features such as drinking, smoking status and LNM status based on CT were classified as no or yes. CEA and CA199 values were recorded as actual measurements. The degree of differentiation was divided into good differentiation and poor differentiation. T stage was recorded according to the 8th edition of the TNM staging system. LNM and distant metastasis were classified as no or yes, respectively.

**Table 1:** Basic information of extracted patients from the First Affiliated Hospital of Nanchang University diagnosed with DPC

Variables	Total	N*_*stage (yes)	N stage (no)	*p* value
Total	224	69	155	
Sex				0.452
Male	128 (57.1)	42 (60.9)	86 (55.5)	
Female	96 (42.9)	27 (39.1)	69 (44.5)	
Age				0.16
< 50	37 (16.5)	15 (21.7)	22 (14.2)	
≥ 50	187 (83.5)	54 (78.3)	133 (85.8)	
Cell differentiation				0.251
Well	57 (25.4)	21 (30.4)	36 (23.3)	
Poorly	167 (74.6)	48 (69.6)	119 (76.7)	
T stage				0.041
T0/T1	19 (8.5)	4 (5.8)	15 (9.7)	
T2	60 (26.8)	11 (15.9)	49 (31.6)	
T3	28 (12.5)	10 (14.5)	18 (11.6)	
T4	117 (52.2)	44 (63.8)	73 (47.1)	
M stage				0.627
M0	189 (84.4)	57 (82.6)	132 (85.2)	
M1	35 (15.6)	12 (17.4)	23 (14.8)	
CEA				0.525
< 6.5	140 (62.5)	41 (59.4)	99 (63.9)	
≥ 6.5	84 (37.5)	28 (40.6)	56 (36.1)	
Smoking				0.389
No	155 (69.2)	45 (65.2)	110 (71.0)	
Yes	69 (30.8)	24 (34.8)	45 (29.0)	
Drinking				0.288
No	157 (70.1)	45 (65.2)	112 (72.3)	
Yes	67 (29.9)	24 (34.8)	43 (27.7)	
Lymph vessel invasion				< 0.001
No	143 (63.8)	28 (40.6)	116 (74.8)	
Yes	80 (35.7)	41 (00.59.4)	39 (25.2)	
Tumour size				0.417
< 2 cm	60 (26.8)	16 (23.2)	44 (28.4)	
≥ 2 cm	164 (73.2)	53 (76.8)	111 (71.6)	
CA199				0.774
< 27	81 (36.2)	24 (34.8)	57 (36.8)	
≥ 27	143 (63.8)	45 (65.2)	98 (63.2)	
CT imaging				< 0.001
No (LNM)	166 (74.1%)	25 (36.2)	141 (90.9)	
Yes (LNM)	58 (25.9%)	44 (36.8)	14 (9.1)	
Survival time (M)	11.0 (6.0, 20.0)	8.0 (5.0, 16.0)	12.0 (6.0, 21.7)	

### CT image acquisition, segmentation, and extraction of radiomics features

**Fig. 1:**
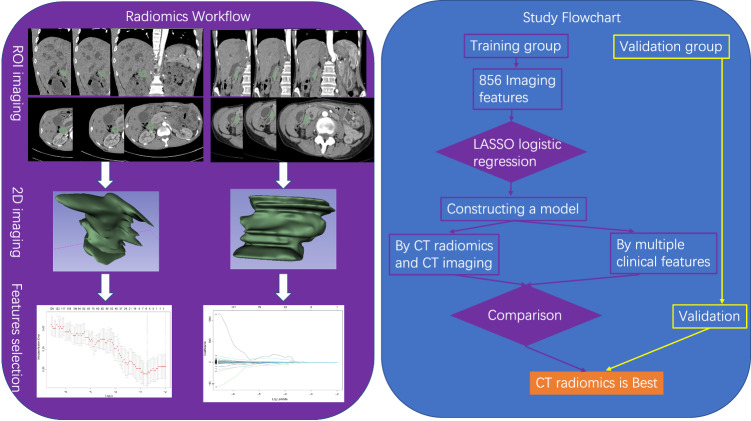
The flowchart of radiomics and the whole study

The radiomics workflow is illustrated in Fig. 1. In this study, all patients underwent contrast-enhanced abdominal CT scans that covered the entire tumour. The CT scans were performed using a Siemens SOMATOM Definition AS 128-slice spiral CT scanner. The CT scan was conducted with the following parameters: 120 kV, 200 effective mAs, a collimation of 640.6 mm, a matrix of 512 × 512, a pitch of 0.8, and a gantry rotation time of 0.5 s. After the nonenhanced CT scan, 80–100 mL of nonionic contrast agent (370 mg I/mL, Pamir iodine, Bracco) was intravenously injected at a rate of 3.5 mL/s, followed by a saline flush (20 mL), after which a dynamic contrast-enhanced CT scan was performed. Images in the arterial phase and venous phase were obtained at 30 s and 60 s, respectively. The slice thickness of the images was 1.0 mm. The CT images in the arterial phase were retrieved for image feature extraction. The region of interest (ROI) for tumour lesions was semiautomatically segmented using 3D Slicer 5.0.3, and the ROI was selected on the slice containing the tumour area. Texture extraction was performed using the radiomics tool 3D Slicer 5.0.3 to extract imaging features from the three-dimensional images of the tumours. ROI segmentation for tumour imaging was conducted by two clinicians with the help of extensively experienced radiologists. Furthermore, intra- and interclass correlation coefficients were calculated to assess the consistency of the two readers in radiomics feature extraction. Distinct radiological characteristics may indicate a suspicious LNM. Non-metastatic LNs typically appear as discrete, kidney-shaped structures composed of soft tissue featuring a concave hilum consisting of fat tissue. In contrast, LNs with metastases appear round on imaging and exhibit rim enhancement, irregular borders, a nonuniform parenchymal staining pattern, and hypodense central attenuation. Additionally, size, typically more than 5 mm, remains a commonly used criterion [16].

### Feature selection and radiomics signature construction

We used the least absolute shrinkage and selection operator (LASSO) logistic regression algorithm, which can achieve dimension reduction for high-dimensional data. A formula, which is shown in Supplementary Material 1, was generated using a linear combination of selected features according to their respective LASSO coefficients; then, the formula was used to determine a risk score (named the CT radiomics model) for each patient to reflect the TNM status of patients with DPC.

### Statistical analysis

For statistical analysis, patients extracted from our centre were first divided into a training group and a testing group at a 1:1 ratio. In addition, features were compared between the two groups. Differences in continuous variables were compared using the Mann‒Whitney *U*-test or independent *t-*test, while comparisons of categorical variables were conducted using the chi-square test or Fisher’s exact test. To construct the model, we simultaneously constructed a radiomics model, a clinical feature model and a CT imaging alone model. For the model’s performance and discriminative ability, we tested the Hosmer–Lemeshow goodness-of-fit test and plotted receiver operating characteristic (ROC) curves to evaluate the model’s classification ability. We used decision curve analysis (DCA) and a clinical impact curve (CIC) to evaluate the clinical net benefit of the predictive models. Additionally, we conducted DeLong’s test to calculate the significant differences in the models. All the statistical analyses were performed using R software, and the relevant software packages were obtained from the R software program website (https://cran.r-project.org/). A *p* value less than 0.05 was considered to indicate statistical significance for all analyses.

## Results

### Basic patient information and study design

In this work, according to the inclusion and exclusion criteria, as shown in Supplementary Fig. 1, we included 224 DPC patients diagnosed in our hospital from January 2018 to September 2022. As shown in Table 1, male patients and older patients (> 50 years) accounted for more than 50% of the DPC patients; however, the imbalance in distribution was not significant in the LNM group. The distribution of cells differentiating between LNM-negative and LNM-positive tumours was similar (*p* > 0.05); however, there were more advanced-stage tumours in the LNM-positive DPC than in the LNM-negative DPC (*p* = 0.041). In terms of distant metastasis, there seemed to be no association with LNM status. Similarly, between the LNM-positive group and the LNM-negative group, the rates of smoking and drinking were similar (*p* > 0.05). Most DPC patients had larger tumours (> 2 cm); however, the size of the tumours was not associated with LNM status. Moreover, the levels of CEA and CA199 did not differ between the LNM-positive and LNM-negative patients. We found that tumours with lympho-vascular invasion tended to be positive for LNM (59.4 vs 25.2, *p* < 0.001). The true positive rate of CT imaging for LNM was not perfect, with a value of 0.745 (44/59). The median survival time of patients with positive LNMs was 8 months (5–16), while patients without LNM had a median survival time of 12 (6–21.7).

### Establishment of a model for predicting LNM in DPC patients

**Fig. 2:**
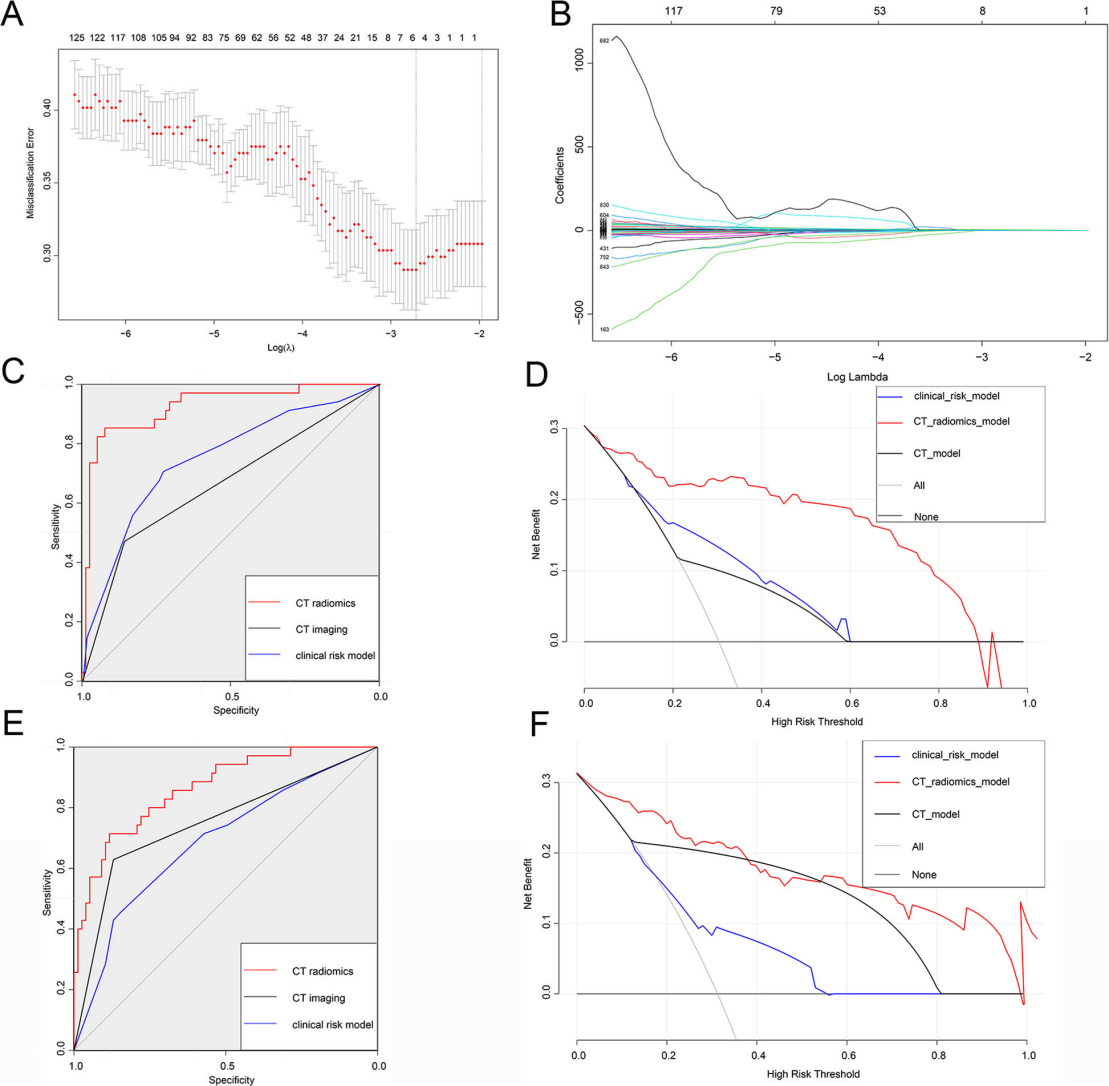
Texture feature selection was determined by LASSO logistic regression and conducted models. **A** Selection of the tuning parameter (λ) according to the LASSO model via 10-fold cross-validation based on minimum criteria. Binomial deviances from the LASSO regression cross-validation procedure were plotted as a function of log(λ). The *y*-axis indicates binomial deviances. The lower *x*-axis indicates the log(λ). Numbers along the upper *x*-axis represent the average number of predictors. Red dots indicate average deviance values for each model with a given λ, and vertical bars through the red dots show the upper and lower values of the deviance. The vertical black lines define the optimal values of λ, where the model provides its best fit to the data. The optimalλ (logλ = −2.75) was selected. **B** LASSO coefficient profiles of the five features. The dotted vertical line was plotted at the value selected using 10-fold cross-validation in (**A**). **C**, **D** ROC and DCA of the CT radiomics model, CT imaging model and clinical risk model predict LNM of DPC in the training group, respectively. **E**, **F** ROC and DCA of the CT radiomics model, CT imaging model and clinical risk model predict LNM of DPC in the training group, respectively

**Fig. 3:**
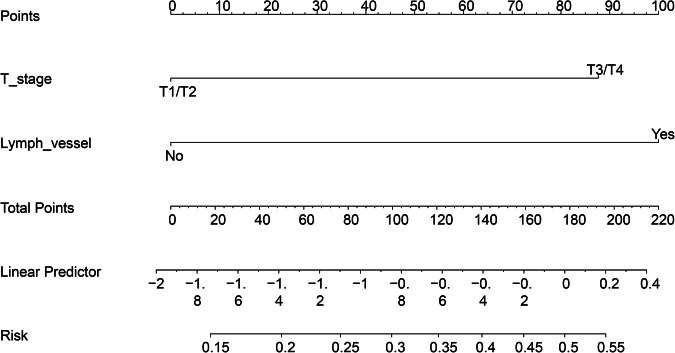
The nomogram constructed by clinical risk factors in our study

Our patients were divided into training and testing groups at a ratio of 1:1. The detailed characteristics of the patients in the training set and testing set are shown in Table 2. There were 112 patients in the training group and 112 patients in the testing group. Table 2 shows that the distribution was random because the *p* value was greater than 0.05. Then, based on the flowchart shown in Fig. 1, we enroled 856 variables, including shape-based variables, first-order statistics and textural features, according to standardised definitions [17]. We performed LASSO logistic regression to identify the features, as shown in Fig. 2A, B. A total of 14 features, which are presented in Supplementary Material 1, were used to construct a model according to the value of λ (logλ = −2.75). Next, we constructed a formula for calculating the radiomics score according to the weight coefficient of the features and constructed a CT radiomics model. Moreover, we performed multivariate logistic regression analysis utilising clinical features, and the results are presented as nomogram plots (Table 3 and Fig. 3). According to the multivariate analysis, T stage and LVI were associated with LNM status, as indicated by a *p* value less than 0.05; early-stage non-LVI was associated with negative LNM (Table 3). Compared to the T stage, LVI contributed the most to LNM (Fig. 3). For the interpretation of the nomogram, each patient had these features, and the risk score was determined according to our nomogram. Next, we constructed a vertical straight line and observed the risk of LNM. Finally, we estimated the risk with a concrete value of accuracy. Furthermore, we constructed a simple model in which CT imaging alone was used to predict the status of LNM.

**Table 2:** Basic information of training group and testing group diagnosed as DPC

Variables	Total	Training group	Testing group	*p* value
Total	224	112	112	
Sex				1
Male	128 (57.1)	64 (57.1)	64 (57.1)	
Female	96 (42.9)	48 (42.9)	48 (42.9)	
Age				0.28
< 50	37 (16.5)	15 (13.4)	22 (19.6)	
≥ 50	187 (83.5)	97 (86.6)	90 (80.4)	
Cell differentiation				0.032
Well	57 (25.4)	21 (18.8)	36 (32.1)	
Poorly	167 (74.6)	91 (81.2)	76 (67.9)	
T stage				1
T0/T1/T2	79 (35.3)	40 (35.7)	39 (34.8)	
T3/T4	145 (64.7)	72 (64.3)	73 (65.2)	
M stage				0.27
M0	189 (84.4)	98 (87.5)	91 (81.2)	
M1	35 (15.6)	14 (12.5)	21 (18.8)	
CEA				0.89
< 6.5	140 (62.5)	71 (63.4)	69 (61.6)	
≥ 6.5	84 (37.5)	41 (36.6)	43 (38.4)	
Smoking				1
No	155 (69.2)	77 (68.8)	78 (69.6)	
Yes	69 (30.8)	35 (31.2)	34 (30.4)	
Drinking				1
No	157 (70.1)	79 (70.5)	78 (69.6)	
Yes	67 (29.9)	33 (29.5)	34 (30.4)	
Tumour size				1
< 2 cm	99 (44.2)	49 (43.8)	50 (44.6)	
≥ 2 cm	125 (55.8)	63 (56.2)	62 (55.4)	
CA199				1
< 6.5	81 (36.2)	41 (36.6)	40 (35.7)	
≥ 6.5	143 (63.8)	71 (63.4)	72 (64.3)	
N stage				1
No	155 (69.2)	78 (69.6)	77 (68.8)	
Yes	69 (30.8)	34 (30.4)	35 (31.2)	
Lymph vessel invasion				0.163
No	144 (64.3)	67 (59.8)	77 (68.7)	
Yes	80 (35.7)	45 (40.2)	35 (31.2)	
CT-positive LNM				0.357
No	166 (74.1%)	85 (75.9)	81 (72.3%)	
Yes	58 (25.9%)	27 (24.1)	31 (27.7)	
Survival time (M)	11.0 (6.0, 20.0)	11.0 (6.0, 18.7)	11.50 (5.0, 21.2)	0.811

**Table 3:** Univariate and multivariate logistic regression model for exploring the potential favourable factors of LNM in DPC patients

Variable		OR (univariable)	OR (multivariable)
Age	< 50	–	
	≥ 50	0.46 (0.18–1.20, *p* = 0.114)	
Sex	Male		
	Female	0.71 (0.31–1.61, *p* = 0.411)	
Differentiation	Low	–	
	High	0.50 (0.22–1.15, *p* = 0.104)	
Size	< 2 cm	–	
	≥ 2 cm	1.57 (0.69–3.55, *p* = 0.283)	
Smoking	No	–	
	Yes	1.08 (0.45–2.55, *p* = 0.868)	
Drinking	No	–	
	Yes	1.30 (0.55–3.07, *p* = 0.543)	
CEA	No	–	
	Yes	1.10 (0.49–2.50, *p* = 0.814)	
CA199	No	–	
	Yes	1.10 (0.47–2.53, *p* = 0.832)	
T stage	T1/T2	–	
	T3/T4	2.84 (1.11–7.31, *p* = 0.030)	2.72 (1.03–7.16, *p* = 0.043)
Lymph vessel invasion	No	–	
	Yes	3.10 (1.33–7.22, *p* = 0.009)	2.98 (1.25–7.08, *p* = 0.013)

### Validation and comparison among the three models

**Fig. 4:**
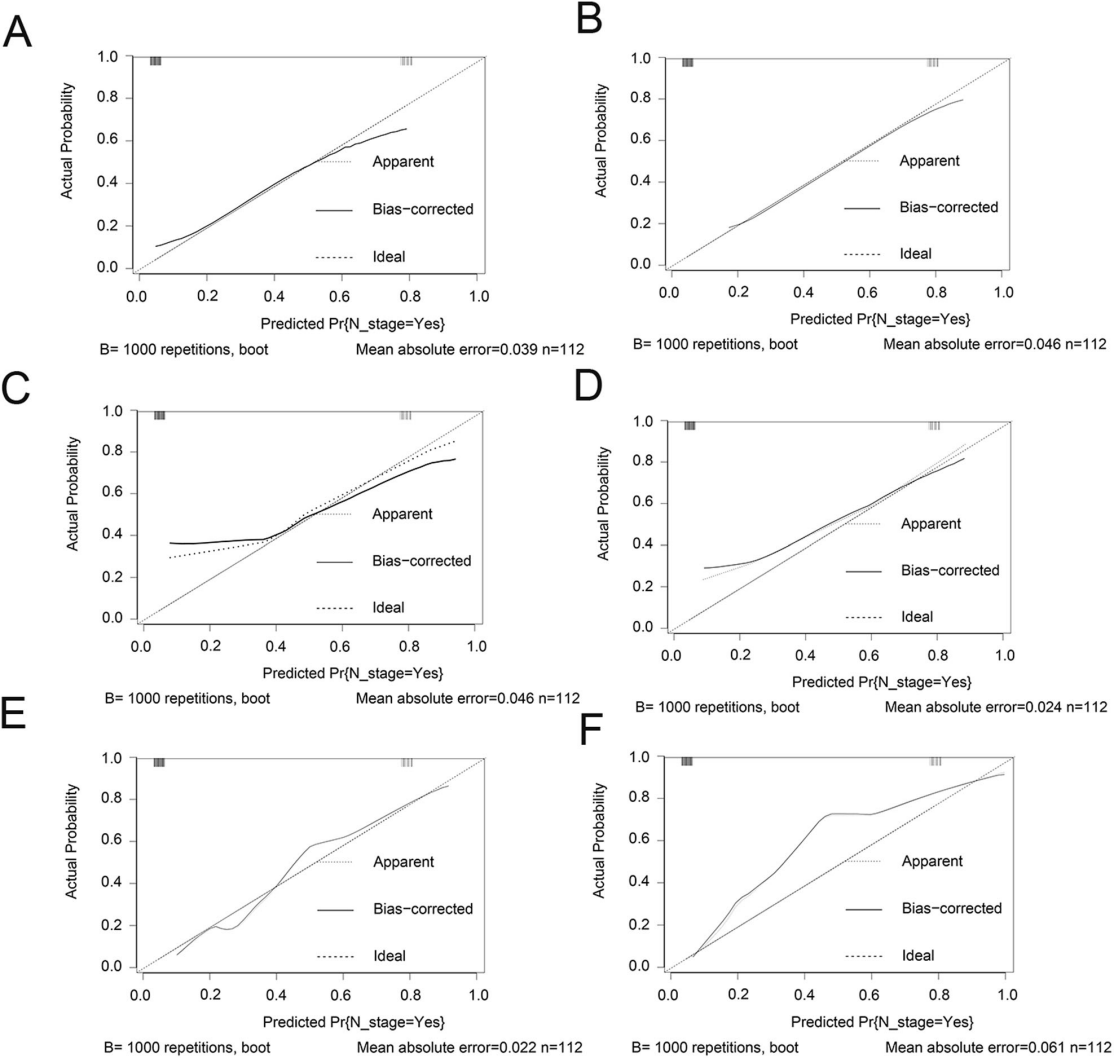
Validation of our models. **A**, **C**, **E** Calibration plot of CT radiomics model, CT imaging model, and clinical risk model in the training group, respectively. **B**, **D**, **F** Calibration plot of CT radiomics model, CT imaging model, and clinical risk model in the testing group, respectively

**Fig. 5:**
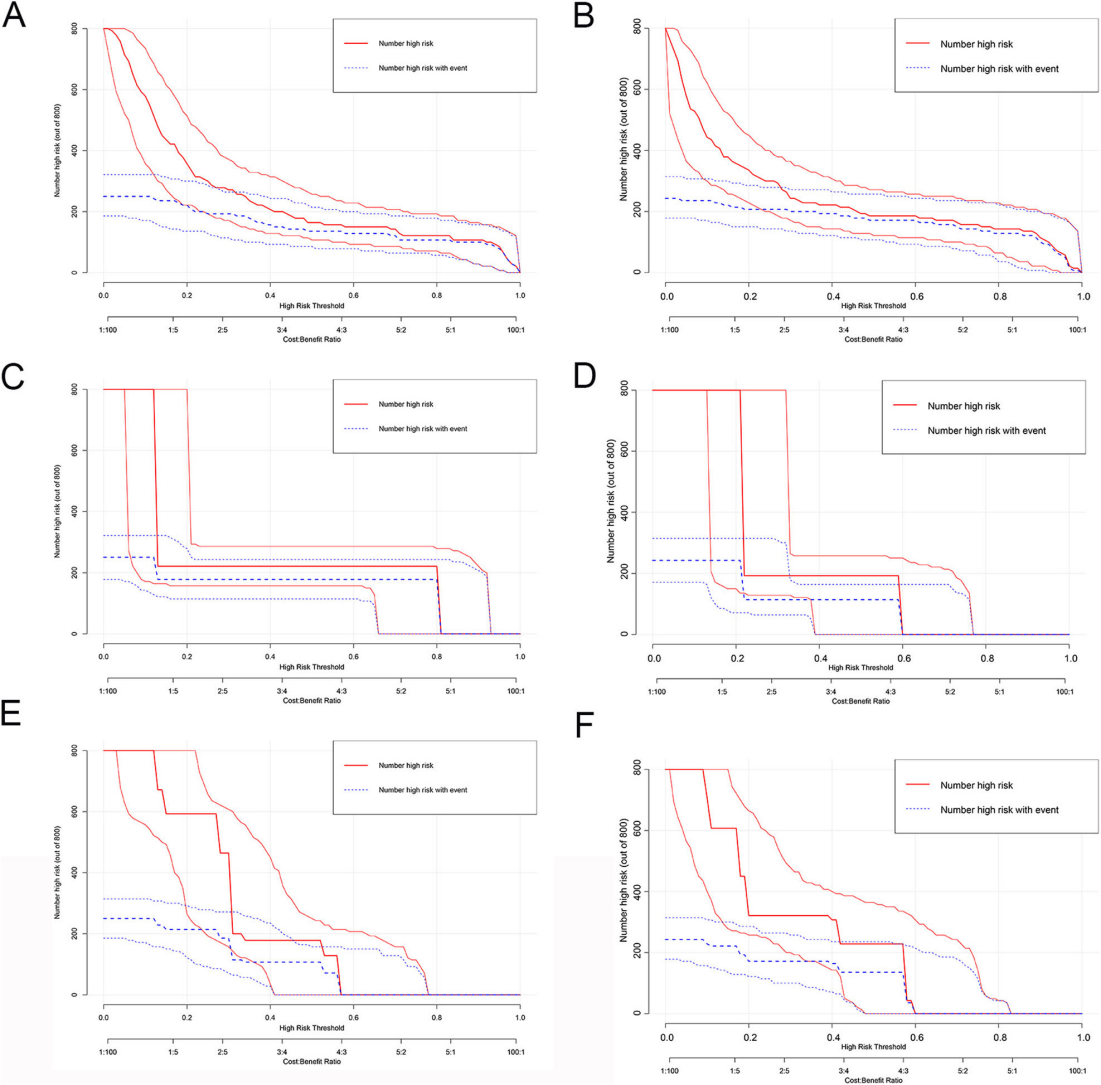
CIC were performed in the training and validation group. **A**, **C**, **E** CIC of CT radiomics model, CT imaging model, and clinical risk model in training, respectively. **B**, **D**, **F** CIC of CT radiomics model, CT imaging model, and clinical risk model in the testing group, respectively

To estimate the performance of our models, first, we performed a ROC analysis on the three models. As shown in Fig. 2C–E, CT radiomics had the highest sensitivity and specificity (AUC = 0.926, 95% CI = 0.869–0.982), followed by CT imaging (AUC = 0.665, 95% CI = 0.571–0.758) and the clinical risk model (AUC = 0.752, 95% CI = 0.65–0.853). The difference between the CT radiomics model and the clinical risk model or CT imaging was significant (*p* < 0.05); however, the difference between the clinical risk model and CT imaging model was not meaningful (Table 4). Similarly, the *Z* score of DeLong’s test showed that the CT radiomics were perfect (Table 4). Similarly, in the testing cohort, the CT radiomics model had the best AUC (AUC = 0.872, 95% CI = 0.802–0.941, *p* < 0.05), while the CT imaging model and clinical risk model had similar performances (AUC = 0.749 vs AUC = 0.0.688, *Z* score = −0.86, *p* = 0.388) (Table 4). As for the clinical effect, Fig. 2D, F, and DCA graphically showed that the use of the CT radiomics model to predict LNM had remarkable predictive power and was superior to the use of the clinical risk model and CT imaging. For the calibration plot, using the bootstrap validation method (*n* = 1000), we observed that the CT radiomics model had good agreement with the actual values (Fig. 4A); however, the other two models had remarkable inconsistencies between the prediction and actual values (Fig. 4C–E). Like in the training cohort, in the testing cohort, the CT imaging model and clinical risk model had poorer consistency between the actual value and the predictive value, while the CT radiomics model remained consistent (Fig. 4B, D, F). Additionally, the CICs of the complex model indicated that the radiomics models had more remarkable predictive power than the other two models in both the training set and the testing set (Fig. 5A–F).

**Table 4:** AUC of ROC for exploring LNM

	AUC value	95% CI	*Z* score	*p* value
Training group				
CT radiomics	0.926	0.869–0.982		
Clinical risk model	0.752	0.65–0.853	2.93 (VS CT radiomics)	0.003
CT imaging	0.665	0.571–0.758	4.67 (VS CT radiomics)	< 0.001
			1.18 (VS clinical risk model)	0.235
Testing group				
CT radiomics	0.872	0.802–0.941	1.21 (VS CT radiomics in training group)	0.053
Clinical risk model	0.688	0.58–0.795	2.81 (VS CT radiomics)	0.005
CT imaging	0.749	0.66–0.839	2.06 (VS CT radiomics)	0.039
			−0.86 (VS clinical risk model)	0.388

### Validation and comparison of the ability of CT radiomics features and clinical risk models to predict patient prognosis

**Fig. 6:**
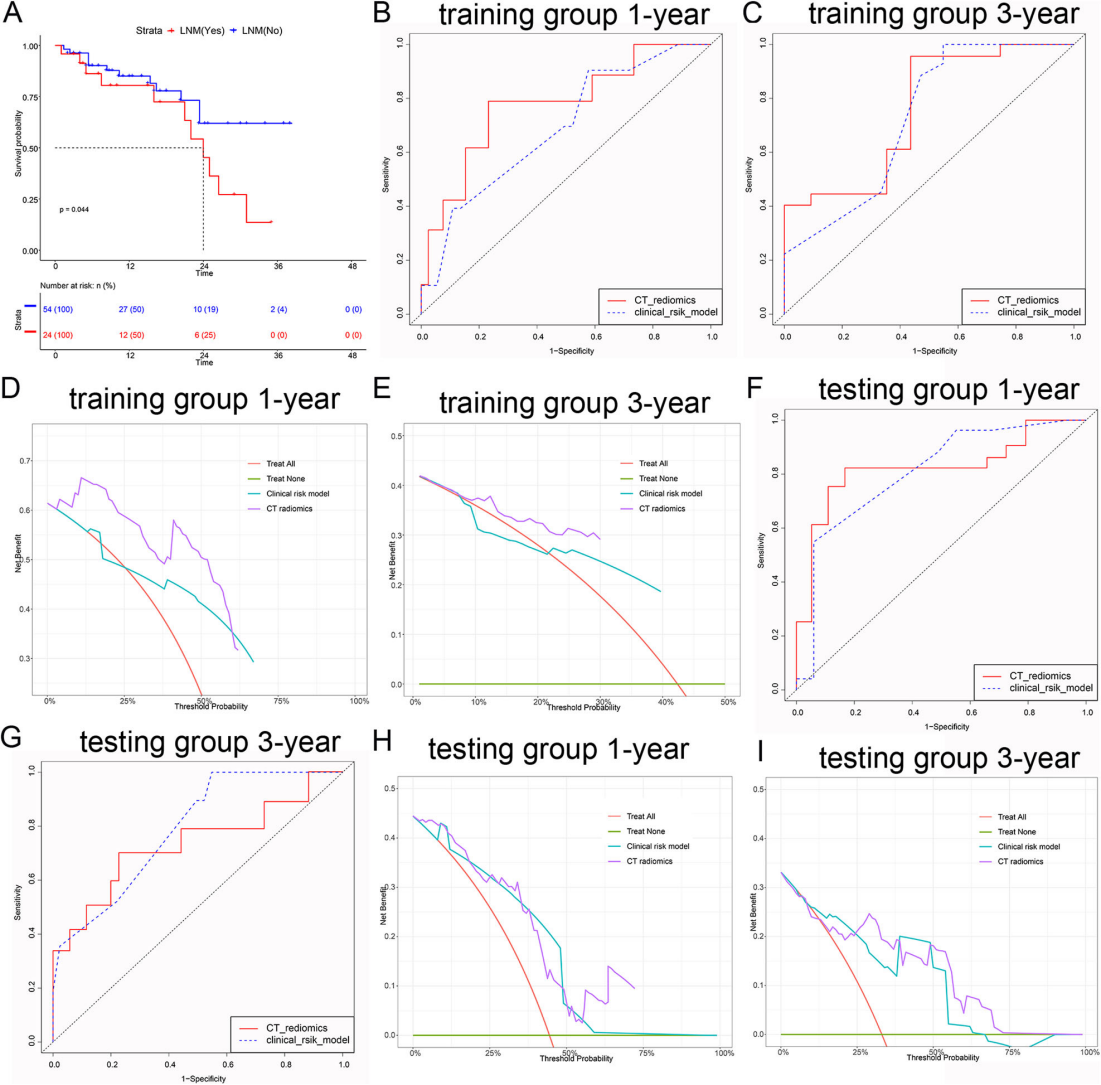
The performance of models was investigated to predict survival by ROC and DCA. **A** K-M survival curve was performed based on the LNM group (positive LNM and negative LNM). **B**, **C** ROC of the CT radiomics model and clinical risk model predicts 1-year and 3-year survival of DPC patients in the training group, respectively. **D**, **E** DCA of the CT radiomics model and clinical risk model predicts 1-year and 3-year survival of DPC patients in the training group, respectively. **F**, **G** ROC of the CT radiomics model and clinical risk model predict 1-year and 3-year survival of DPC patients in the testing group, respectively. **H**, **I** DCA of CT radiomics model and clinical risk model predicts 1-year and 3-year survival of DPC patients in the testing group, respectively

Previously, our models exhibited good performance for LNM prediction; however, whether our model was effective at predicting patient prognosis was unknown. First, we generated K‒M survival curves to evaluate the association between LNM and survival. Figure 6A shows that patients with positive LNM had poorer survival than did those without LNM (*p* = 0.044). Then, we evaluated the efficacy of the models for predicting survival. As shown in Fig. 6B, C, in the training cohort, CT radiomics had high sensitivity and specificity for predicting 1-year and 3-year survival (1-year, AUC = 0.753, 95% CI = 0.711–0.821; 3-year, AUC = 0.661, 95% CI = 0.612–0.732), while the clinical risk factor model had a smaller AUC for predicting 1-year survival and 3-year survival (1-year, AUC = 0.703, 95% CI = 0.653–0.758; 3-year, AUC = 0.643, 95% CI = 0.609–0.719); however, only the difference in predicting 1-year survival was significant (*p* = 0.046). As for the clinical effect, Fig. 6D, E, and DCA graphically showed that the CT radiomics model had greater predictive power than the clinical risk model. In the testing cohort (Fig. 6F–I), regardless of the ROC curve or DCA, the CT radiomics model and clinical risk model had remarkable power for predicting survival. The difference between them was not statistically significant (Table 5).

**Table 5:** Accuracy of the prediction model for estimating the prognosis of patients with DPC

Variable	Value (95% CI)			
	Training group	*p* value	Testing group	*p* value
1-year AUC for CT radiomics	0.753 (0.711–0.821)	0.046	0.751 (0.705–0.838)	0.091
1-year AUC for clinical risk model	0.703 (0.653–0.758)		0.713 (0.644–0.801)	
3-year AUC for CT radiomics	0.661 (0.612–0.732)	0.327	0.653 (0.549–0.707)	0.162
3-year AUC for clinical risk model	0.643 (0.609–0.719)		0.671 (0.615–0.737)	

## Discussion

The incidence of DPC transformation from adenoma to cancer ranges from 25% to 85% [18]. Moreover, surgery remains the primary treatment strategy for patients with DPC, but the extent of surgical intervention is difficult to control because the status of LNM is difficult to diagnose [19]. Medical imaging has greatly advanced cancer diagnosis and treatment planning with the emergence of ‘radiomics’, a field that involves high-throughput data mining of medical images [20]. In our study, we constructed a radiomics model via LASSO and analysed its performance in predicting LNM and survival; the radiomics model for assessing LNM had the best predictive performance.

Previous studies have demonstrated that CT imaging is helpful for the diagnosis of duodenal papilla disease, but the traditional CT features of patients with DPC tend to be similar to those of patients with chronic mucositis except for larger tumours, easily leading to misdiagnosis [21]. Clinically, CT images evaluate the status of LNM based on size and morphology and are considered to indicate a lesion when the size of the lymph node is greater than 5 mm in diameter [19]. In our study, we found that the area under the curve (AUC) of CT for the diagnosis of LNM ranged from 0.665 to 0.749, which is consistent with the findings of previous studies [21, 22]. Hence, to improve the diagnosis of DPC and its TNM stage, comprehensive diagnosis should include a combination of other clinical characteristics and new technologies. To our knowledge, few studies have constructed models to predict LNM and survival in patients with duodenal malignant tumours via radiomics analysis [23]. Previous studies have shown that T stage and lymph vessel invasion are independent risk factors for LNM [24–26]. Our results are consistent with the above studies, and it is reasonable that surgery should be performed for DPC in advanced stages. Radiomics, an emerging image quantification approach, has been widely used in the diagnosis and prognosis of cancer based on medical images. Some studies used support vector machines and other deep learning methods, while our study applied LASSO regression analysis to select potent variables, both of which could improve efficiency. As radiomics involves the use of large amounts of medical image data, efficient methods are needed to extract relevant information from these large radiomics datasets. Hence, due to the ability to fully utilise data, radiomics often compensates for the shortcomings of traditional CT. As expected, our radiomics model exhibited the best performance in predicting LNM, which was also in line with the findings of previous studies [15, 27].

The association between LNM and survival is strong, in which the risk of cancer-specific death seems to be two to three times greater; moreover, LNM is strictly related to increased cancer recurrence and worse oncological outcomes [1]. In line with these findings, we found that patients with positive LNM had poorer survival, and most patients with LNM were in the T3/T4 stage [28]. Previous studies reported that the 5-year overall survival (OS) rate of patients with DPC ranged from 30% to 70% [1, 28], but our data showed that the 3-year OS rate was low. Previous models for predicting duodenal cancer prognosis were built with clinical risk factors, the C-index of which ranged from 0.6 to 0.7 [10, 29, 30]. In our study, the model for predicting survival had an AUC of more than 0.7 for 1-year or 3-year survival, which also showed good performance compared to that of the other models. Nevertheless, our findings suggest that the radiomics model did not show a discernible edge over our clinical model. It is plausible that the restricted sample size may have contributed to this outcome.

Nevertheless, our study has several limitations. First, our study was a single-centre retrospective study that included only 224 DPC patients, decreasing the reliability and possibility of popularising the findings. Hence, further study is needed to validate the performance and generalizability of our models to other populations. Second, the included clinical risk factors were limited, resulting in unreliable clinical risk models. Third, in this work, we focused only on clinical risk factors, ignoring the potential genetic markers involved. Finally, the radiomics analysis in our study was based on images of the primary tumours rather than on the lymph nodes. In fact, there are still few studies that establish a radiomics model based on lymph nodes for LNM prediction in DPC patients, and we think it would be more reliable to evaluate the efficacy of predicting LNM compared to traditional CT. Future research is needed to explore the feasibility and predictive value of radiomics analysis based on lymph node imaging or a combination of primary tumour and lymph node images.

## Conclusion

On the whole, as evidenced by results from the training and testing groups, our radiomics model demonstrated superior performance in predicting LNM compared to both the standalone CT imaging model and the clinical risk model. However, further studies are needed to explore whether the radiomics model is superior to the model based on clinical risk factors. In the future, we envisage that radiomics models have the potential to transform the screening of DPC patients and subsequently contribute to DPC management.

### Supplementary information

ELECTRONIC SUPPLEMENTARY MATERIAL

## Data Availability

All data generated or analysed during this study are included in this published article and its supplementary information file.

## Abbreviations

CIC: Clinical impact curve
DCA: Decision curve analysis
DPC: Duodenal papillary carcinoma
LASSO: Least absolute shrinkage and selection operator
LNM: Lymph node metastasis
LVI: Lympho-vascular invasion
OS: Overall survival
ROC: Receiver operating characteristic
