# Supplementary material for: Radiomics analysis based on CT for predicting lymph node metastasis and prognosis in duodenal papillary carcinoma
Source: Insights Imaging. 2024 Jun 20;15:155. doi: 10.1186/s13244-024-01732-6 (PMC11190116; doi:10.1186/s13244-024-01732-6)
Supplement: Supplementary file 1 — ELECTRONIC SUPPLEMENTARY MATERIAL [file 13244_2024_1732_MOESM1_ESM.pdf]

Radiomics analysis based on CT for predicting lymph node metastasis and prognosis in duodenal papillary carcinoma

ELECTRONIC SUPPLEMENTARY MATERIAL

Supplementary Material 1: Formula generated using a linear combination of selected features according to their respective LASSO coefficients.

LNM_P risk score=-1.345007e^{01}+2.4865e^{-04}*Maximum2DDiameterColumn-7.538659e-03*Skewness - LargeAreaLowGrayLevelEmphasis*4.814778e^{-06} - LowGrayLevelZoneEmphasis.1*1.253950 - SmallAreaLowGrayLevelEmphasis*(1.741708e^{-01}) + ClusterProminence*(4.162495e^{-02})+LowGrayLevelZoneEmphasis.3*(2.233884)+SmallAreaLowGrayLevelEmphasis.3*(2.330414e^{-02})+SmallAreaLowGrayLevelEmphasis.4*(-1.041390)+ZoneEntropy.5*(-1.877607e^{-01})+Kurtosis.6*(4.247290e^{-02})+SmallAreaEmphasis.6*(4.590376e^{-08})+DifferenceEntropy.7*(1.258183e^{1})+GrayLevelNonUniformityNormalized.15*(-1.530301e^{-01})

Supplementary Figure 1: Study flowchart

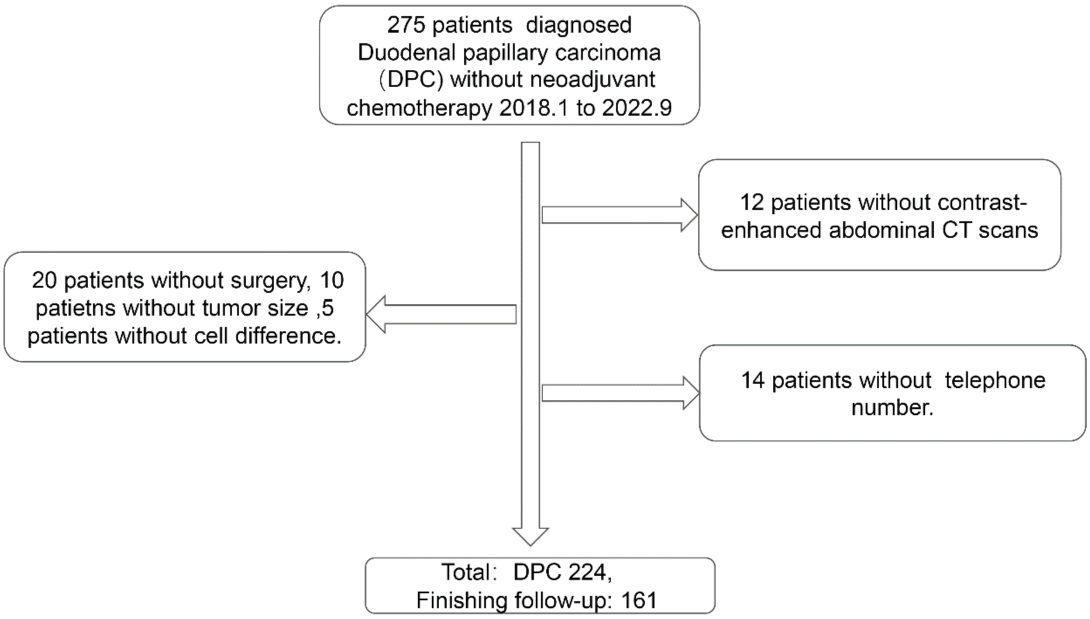

Insights Imaging (2024) Tang CT, Wu Y, Jiang L, Zeng CY, Chen YX.
